# Association Between Plant-Based Diet Quality and Metabolic Dysfunction-Associated Steatotic Liver Disease (MASLD) in the Multiethnic Cohort Study

**DOI:** 10.21203/rs.3.rs-7403556/v1

**Published:** 2025-09-09

**Authors:** Darya Moosavi, Unhee Lim, Meredith Hullar, Christoph Rettenmeier, Sandi Kwee, Kristine Monroe, Thomas Ernst, Tim Randolph, Lynne Wilkens, Loic Le Marchand, Johanna Lampe, Song-Yi Park

**Affiliations:** Fred Hutch Cancer Center; University of Hawaii at Manoa; Fred Hutchinson Cancer Center; Keck School of Medicine, and Norris Comprehensive Cancer Center, University of Southern California; Keck School of Medicine, and Norris Comprehensive Cancer Center, University of Southern California; Keck School of Medicine, and Norris Comprehensive Cancer Center, University of Southern California; University of Hawaii Cancer Center; University of Hawaii Cancer Center; University of Hawaii Cancer Center; University of Hawaii Cancer Center; Fred Hutchinson Cancer Research Center; University of Hawaii

**Keywords:** MASLD, liver fat, healthful plant-based diet

## Abstract

**Background:**

Metabolic dysfunction-associated steatotic liver disease (MASLD), formerly non-alcoholic fatty liver disease (NAFLD), is a growing public health concern with limited effective treatments. Diet quality may influence MASLD risk, yet the role of plant-based diet quality across diverse populations remains unclear.

**Objective:**

To evaluate the associations of plant-based dietary patterns with liver fat content or MASLD prevalence in multiethnic older adults.

**Methods:**

We analyzed cross-sectional data on 1,598 participants in the Adiposity Phenotype Study (APS), nested within the Multiethnic Cohort Study. Scores for three established plant-based diet indices were computed from food frequency questionnaire responses: overall plant-based diet index (PDI), healthful plant-based diet index (hPDI), and unhealthful plant-based diet index (uPDI). Liver fat was measured using MRI, and MASLD was defined, among participants reporting zero to low alcohol intakes. Multivariable linear models of liver fat and logistic models of MASLD were used to estimate their associations with the plant-based diet indices, adjusting for demographic, lifestyle, and anthropometric covariates.

**Results:**

Higher hPDI scores were associated with lower liver fat content (adjusted mean for 4th (5.39) vs. 1st quartile (6.52) and reduced likelihood of MASLD (OR for 4th vs. 1st quartile = 0.58 (95% CI: 0.41–0.81). When stratified across five racial and ethnic groups, stronger inverse associations were observed among Latino and White participants (p-heterogeneity = 0.001) than among African American, Japanese American, or Native Hawaiian participants. No consistent associations were observed for PDI or uPDI. Among hPDI components, higher nut and lower animal fat intakes were associated with lower liver fat and MASLD.

**Conclusions:**

Greater adherence to a healthful plant-based diet is associated with lower liver fat and MASLD prevalence, with some racial and ethnic variation. These findings underscore the importance of plant-food quality and may inform dietary strategies for MASLD prevention in heterogeneous populations.

## Introduction

Metabolic dysfunction-associated steatotic liver disease (MASLD), formerly known as non-alcoholic fatty liver disease (NAFLD), is a growing global health concern with substantial implications for chronic liver disease and cancer.^1^ The prevalence of MASLD is high; under the previous definition of NAFLD, the prevalence was 30% worldwide and has increased by 50% over the past two decades.^2^ MASLD is now the primary cause of chronic liver disease and liver cancer, due to recent preventive and therapeutic advances for chronic viral hepatitis.^3,4^ It is also a significant contributor to the risk of non-hepatic cancers and premature mortality from cardiometabolic diseases, likely because of the critical role of the liver in systemic health.^5–8^ Considering the expansive impact of MASLD and the lack of effective pharmacologic treatments, lifestyle approaches that can prevent or reverse MASLD are of paramount importance.^9^

Critical lifestyle approaches against MASLD include weight loss, regular exercise, and a healthy diet.^10,11^ Weight loss has been associated with regression of non-alcoholic steatohepatitis (NASH) and liver fibrosis.^10–12^ Diet plays a crucial role in the development and progression of MASLD, with specific dietary patterns linked to the disease independent of weight change. Consuming higher amounts of animal products, soft drinks, and total fat has been found to increase the risk of MASLD.^13–17^ Conversely, plant-based diets have been inversely related to MASLD.^15,16,18,19^ Diets rich in whole grains, vegetables, legumes, and nuts are associated with improved insulin sensitivity, reduced adiposity, and lower systemic inflammation.^20–22^ However, not all plant-based diets are nutritionally equivalent. Differentiating between healthful plant-based diets, characterized by whole plant foods, and unhealthful plant-based diets, characterized by refined grains, sugary beverages, and sweets, provides a necessary, nuanced understanding of plant-based diets to assess their true impact on health.^20^ Consistently, studies have shown that unhealthful plant-based diets are unfavorably associated with liver cancer,^23–25^ while overall and healthful plant-based diets have been linked to varying health impacts.^20,26–29^

To address this gap, we examined liver health in association with three established plant-based diet indices: the overall plant-based diet index (PDI), the healthful plant-based diet index (hPDI), and the unhealthful plant-based diet index (uPDI).^20^ We used data from the Adiposity Phenotype Study (APS), nested within the Multiethnic Cohort Study (MEC), to explore the relationships in a diverse population known to have a wide range of susceptibility to liver fat deposition,^30^ as well as large dietary variation.^31^ We hypothesized that PDI and hPDI are inversely correlated with MASLD status, while uPDI is positively correlated, and aimed to identify contributing component foods.

## Materials and Methods

### Study population.

The MEC is an ongoing prospective cohort study, established in Hawaii and southern California with a population-based sample of over 215,000 individuals, aged 45–75 at enrollment (1993–1996) and primarily from five self-identified racial/ethnic groups (Japanese American, Native Hawaiian, White, African American, and Latino).^32^ The APS study was conducted in 2013–2016 among 1,861 MEC participants, aged 60–77, to compare body composition and body fat distribution across the five race/ethnic groups in the MEC, to understand the groups’ heterogeneous BMI-disease associations.

To ensure a wide BMI representation and comparability across sex-racial/ethnic groups, participants were recruited into reasonably balanced strata by sex, race/ethnicity, and six BMI categories (between18.5 and 40 kg/m^2^). Also, to explore the ‘omics biomarker relationship with body fat distribution in physiologic homeostasis, the study excluded individuals who had advanced diseases (e.g., insulin-dependent diabetes, thyroid disorder on medications, or end-stage renal disease), recent smoking history (< 2 years), or chronic viral hepatitis.^30^ The participants underwent a whole-body DXA (dual-energy X-ray absorptiometry) and abdominal MRI scan, as well as anthropometric measurements, fasting blood and stool sample collections, and questionnaires. The study protocol was approved by the institutional review boards at the University of Hawaii (UH) and the University of Southern California (USC), and all participants provided informed consent.

### Construction of plant-based diet indices.

Participants filled out the MEC quantitative food frequency questionnaire (QFFQ), specifically developed for the diverse population and validated against multiple 24-hour recalls.^33^ Based on their reported intake frequency and portion size of over 180 food items in the QFFQ during the previous year, nutrient and energy intakes were estimated using the food composition tables established at the UH Cancer Center.

Briefly, plant-based diet scores, including PDI, hPDI, and uPDI, were calculated using data from the APS QFFQ, following the established scoring algorithms.^20^ These indices are based on the same 18 predefined food groups categorized as healthy plant foods (whole grains, fruits, vegetables, nuts, legumes, vegetable oils, tea/coffee), less healthy plant foods (refined grains, sugar-sweetened beverages, sweets/desserts, potatoes, fruit juices), and animal-based foods (dairy, meat, eggs, fish/seafood, animal fats, miscellaneous animal-based foods).^20,26^ In the MEC and APS, we modified the original 18 food groups by combining sugar-sweetened beverages and sweets/desserts into a single “added sugars” group and excluded miscellaneous animal-based foods.^27^ These modifications were made to fit the MyPyramid Equivalents Database (MPED) used in the MEC for standardizing various foods consumed in different amounts per serving. The MPED disaggregates complex foods into individual ingredients and classifies them into 32 USDA-defined food groups expressed as cup or ounce equivalents. For items not covered by MPED, such as vegetable oils, tea/coffee, and animal fats, gram-based intakes were used instead.

The intake amount for each food group (MPED units or grams per 1000 kcal/day) was ranked into sex-specific quintiles, and the increasing quintiles (from 1st to 5th quintile) were scored differently depending on the index. For PDI, increasing quintiles of all plant foods were scored positively (1 to 5), whereas all animal foods were scored inversely (5 to 1). For hPDI, increasing quintiles of healthy plant foods were scored positively, and less healthy plant foods and all animal foods were scored inversely. For uPDI, increasing quintiles of less healthy plant foods were scored positively, and healthy plant foods and animal foods were scored inversely. For each index, the possible range of the total score was between 16 and 80 (scoring 1 and 5, respectively, for all 16 food groups). All indices were adjusted for total energy intake, as noted above, to reduce confounding and minimize measurement error.

### Assessment of liver fat and MASLD.

Liver fat, in percent volume, was measured using a Dixon-type triple-echo gradient echo MRI protocol.^34^ Participants were classified as cases or non-cases for MASLD according to the definition.^1^

## Statistical Analysis

Of the 1,861 in the APS, the current analysis excluded 229 participants with high reported alcohol intake (men > 30g/day, women > 20g/day) according to the MASLD criteria and 34 participants with missing dietary data, resulting in a final sample of 1,598 participants in the analysis (Supplementary Fig. 1). As shown in Supplementary Table S1, there was no substantial difference between the entire APS data and the analysis subset. Liver fat values, right-skewed, were log-transformed in order to meet the linear regression assumption. Plant-based dietary indices (PDI, hPDI, and uPDI) were categorized into quartiles using the cut-points among participants without MASLD. Analyses were conducted using a complete-case approach.

All analyses were performed using R (version 4.3.0), using two-sided tests with a significance threshold of α = 0.05. Participant characteristics were examined based on means and standard deviations for continuous variables, and frequencies and percentages for categorical variables. Multivariable linear regression (beta coefficients and 95% confidence intervals (CIs)) and analysis of covariance models (adjusted means and 95% CIs) were used to evaluate associations between dietary index quartiles and liver fat. Assumptions of linearity, homoscedasticity, and normally distributed residuals were assessed using diagnostic plots. For the associations between plant-based diet indices and MASLD in odds ratios (ORs) and 95% CIs, multivariable logistic regression models were fitted. Model assumptions, including multicollinearity, model fit, and influential observations, were assessed through variance inflation factors, residual diagnostics, and deviance statistics.

The base model for linear and logistic regression included age, sex, and race/ethnicity, and further adjustment was considered for: (1) physical activity (metabolic equivalents/day), alcohol intake (g/day), and smoking history (never, former smoker) as potential confounders of dietary patterns; (2) BMI to examine the diet associations with hepatic adiposity independently of overall adiposity; and (3) total energy intake (kcal/day) for any residual confounding. To avoid overfitting, covariates that did not change the main diet-liver outcome association were not retained. As a result, the final models included age, sex, race/ethnicity, BMI, and physical activity, where liver fat showed large variations by race/ethnicity as previously reported^30^ and was associated positively with BMI and negatively with physical activity. The models also accounted for a significant interaction between sex and BMI on liver fat or MASLD.

Heterogeneity in the association between plant-based diet indices and liver fat or MASLD was examined by including an interaction term between the specific plant-based diet index and the potential modifiers (sex, race/ethnicity, and BMI). For significant interactions detected, stratified results are presented.

Additional analyses explored the contribution of the 16 individual food groups that provided the component scores, based on quintiles as described above, to all three indices. Mean scores of each component were calculated, with 1 representing the lowest intake and 5 representing the highest for all food groups. Multivariable linear models of log-transformed liver fat and logistic models of MASLD were then fitted to examine their association with the 16 food group components, adjusting for the same covariates as in the primary models.

## Results

### Participant characteristics.

The characteristics of the 1,598 participants are presented across the extreme quartiles of the plant-based diet indices in Table 1. The mean age (69.2 years overall) and the sex distribution (52% women overall) were generally consistent across the adherence quartiles of all three indices; however, the racial/ethnic composition differed. For PDI and uPDI, the highest quartile (Q4), compared to the lowest quartile (Q1), was more likely to have African American or Latino participants, whereas Q4 for hPDI had more Japanese American, Latino, or White participants. PDI and uPDI were also similar in having more former smokers in Q4 compared with Q1, while smoking history was similar across the hPDI quartiles. Higher adherence (higher quartiles) to both PDI and uPDI was associated with lower BMI and greater physical activity, while the opposite trend was seen with uPDI. Across the quartiles of all three indices, the variation in alcohol intake was small among these participants who reported no or low consumption.

### Association of plant-based diet indices with liver fat and MASLD.

The association of plant-based diet indices (PDI, hPDI, uPDI), each in quartiles, with liver fat content is presented in Table 2, and the association with MASLD in Table 3. No significant association was observed between PDI and liver fat or MASLD. In contrast, higher adherence to a healthful plant-based diet was significantly associated with lower liver fat content. Participants in the 3rd and 4th quartiles of hPDI had significantly lower liver fat levels (5.96% and 5.39%, respectively) compared to the lowest quartile (6.52%), with a borderline significance for 2nd quartile (6.19%). For uPDI, no consistent association was observed across the upper quartiles. Similarly, the association with MASLD was only significant for hPDI (Table 3). Participants in higher quartiles for hPDI showed lower odds of having MASLD, with up to 42% lower odds for Q4 compared to those in Q1 (OR = 0.58; 95% CI: 0.41–0.81) (p-trend = 0.002).

### Race/ethnicity-specific associations of hPDI with liver fat or MASLD.

**Figure 1:**
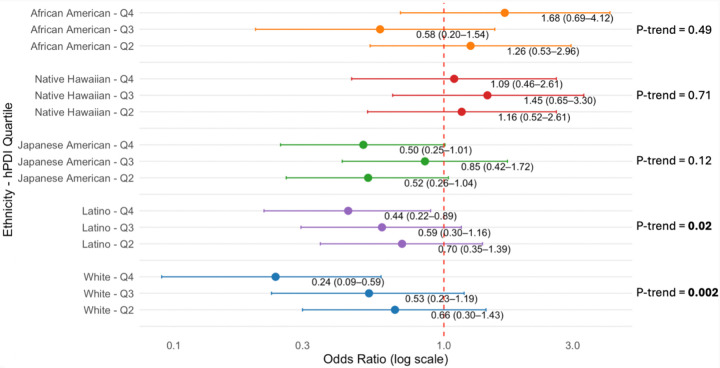
Association between the healthful plant-based diet index (hPDI) and metabolic dysfunction–associated steatotic liver disease (MASLD), stratified race/ethnicity. Odds ratios for upper quartiles (closed circles) and 95% confidence intervals (bars) in reference to Quartile 1 (dashed line for odds ratio = 1) are plotted in each racial/ethnic group. The logistic models for the groups are adjusted for age, sex, BMI, and physical activity.

hPDI showed heterogeneous relationships across the racial/ethnic groups to continuous levels of liver fat (*p* for interaction = 0.01) and to the likelihood of MASLD (p-interaction = 0.01). Thus, we present stratified associations for liver fat (Supplementary Table S2) and for MASLD (Fig. 1). Significant inverse associations were observed only among Latino and White participants. The adjusted mean liver fat for the lowest hPDI quartile was higher in Latino participants (6.40%) than in Whites (4.46%), but both groups showed significantly lower liver fat associated with the highest hPDI quartile (4.72% and 3.30%, respectively). No significant associations or consistent trends were observed in Black, Native Hawaiian, or Japanese American participants across hPDI quartiles for liver fat.

Similarly differential associations were detected between hPDI and MASLD across racial/ethnic groups (Fig. 1), showing stronger inverse association only among Latino and White participants. In both groups, the highest quartile adherence to hPDI was associated with a lower likelihood of having MASLD (OR compared to Q1 = 0.44 (0.22–0.89; p-trend = 0.02) for Latinos; OR = 0.27 (0.12–0.61; p-trend = 0.002). In African American, Native Hawaiian, and Japanese American participants, no significant quartile-level associations or overall trends were observed.

### Component food groups by race/ethnicity.

**Figure 2:**
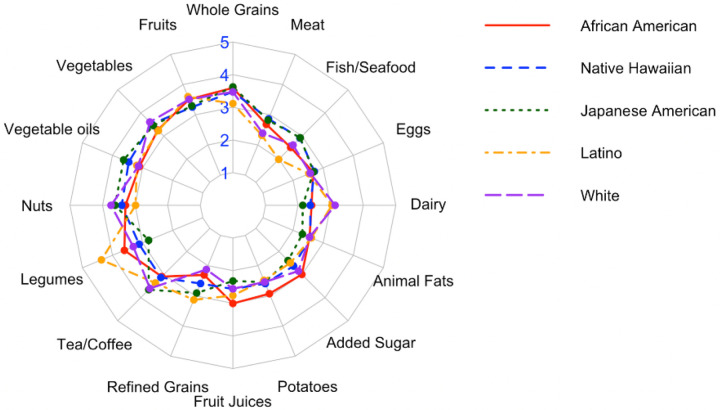
Radar plot of plant-based diet index component food scores across ethnic groups. Each axis represents one of the 16 component food groups. Values range from 1 to 5, with higher scores indicating greater intake.

Figure 2 shows the distribution of hPDI component food scores by race/ethnicity in a radar plot, with 1 representing the lowest intake and 5 representing the highest intake. There was notable variation in component food intakes by ethnicity. Latino participants had the highest average intake of legumes, while Japanese American participants reported slightly higher fish/seafood intake compared to other groups. White participants had relatively greater intake of nuts, dairy and tea/coffee, and the lowest intake of refined grains. African American participants had lower intakes for tea/coffee and vegetable oil, and higher intakes of added sugar, potatoes, and fruit juices. Native Hawaiian participants showed a moderately high intake of eggs and a lower consumption of tea/coffee. Across groups, intake of core plant-based foods (e.g., fruits, vegetables, whole grains) was relatively consistent, while intake of discretionary and animal-derived foods varied more widely by ethnicity.

### Association of component food groups for plant-based diet indices with liver fat or MASLD.

**Figure 3:**
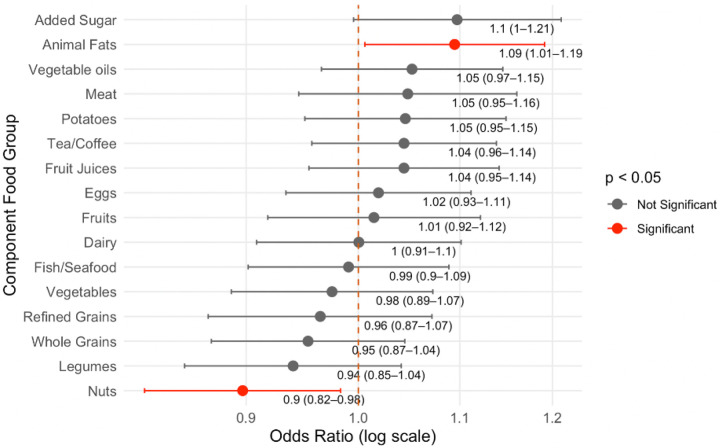
Adjusted associations between individual component scores and MASLD. Odds ratios (OR) per one-point increase and 95% confidence intervals (CI) were estimated using multivariable logistic regression, adjusted for age, sex, race/ethnicity, BMI, and physical activity. The red dashed vertical line at OR = 1.0 represents the null value.

In a multivariable logistic regression model, the associations of individual component food scores of the plant-based diet indices with MASLD were evaluated (Fig. 3, Supplementary Table S3). Of the 16 component food groups analyzed, nut intake was significantly associated with lower odds of MASLD (OR per one-point increase = 0.90, 95% CI: 0.82–0.98). Conversely, higher intakes of animal fat were associated with higher MASLD risk (OR = 1.09, 95% CI: 1.01–1.19, p = 0.04). Other components, including whole grains, fruits, vegetables, legumes, and added sugar, did not show significant independent associations with MASLD, although added sugar approached borderline significance (OR = 1.1, 95% CI:1.00–1.21, p = 0.06). Similar associations with liver fat content are presented in Supplementary Table S4.

## Discussion

In this cross-sectional analysis of the MEC-APS, we examined the associations between three plant-based diet indices (PDI, hPDI, uPDI), and both MRI-measured liver fat content and MASLD status. We found that greater adherence to a healthful plant-based diet was significantly associated with lower liver fat levels and reduced odds of MASLD. This inverse association appeared strongest among Latino and White participants, and a weaker, borderline-significant trend was also noted in Japanese Americans. These findings emphasize the potential importance of the quality of plant foods on liver fat accumulation and suggest that the benefits of healthful plant-based diets may vary by race and ethnicity.

Our results are in line with a growing body of literature linking diet quality to liver-related outcomes. A prospective study from the MEC reported that higher hPDI scores were inversely associated with hepatocellular carcinoma (HCC) risk, particularly among Asian and White participants.^22^ In a cross-sectional analysis of NHANES 2005–2010 data, Li et al. found that greater adherence to both the PDI and hPDI was associated with lower odds of NAFLD and more favorable liver function markers, including ALT, AST, and the Fatty Liver Index (FLI).^35^ Similarly, Mazidi et al. reported associations between higher hPDI and lower odds of NAFLD, also using FLI and biochemical indicators in NHANES participants.^18^ Our study extends these findings by focusing on MASLD and liver fat, and by incorporating objective imaging-based measures rather than surrogate indices.

In contrast to the consistent inverse associations observed with hPDI, we did not observe clear or significant associations between uPDI and MASLD outcomes. This finding differs from prior studies using NHANES data showing a significant positive association between higher uPDI scores and increased NAFLD risk in nationally representative U.S. samples.^18,35^ For example, Li et al. found that individuals in the highest uPDI tertile (≥ 55) had 37% higher odds of NAFLD compared to the lowest (≤ 49), using a uPDI score range of 27–78 and a sample of 6,292 adults. In comparison, our study included a slightly narrower uPDI score range (26–70) and a smaller sample size (~ 1,700), which may limit power to detect modest associations. Additionally, while Li et al. used tertiles, we applied quartiles derived from the distribution of non-cases, yielding fairly even cutoffs. Despite applying these data-driven cutoffs, we did not observe a monotonic trend across quartiles. Interestingly, our findings showed a statistically significant inverse association in Q3 of uPDI and liver fat, but no consistent trend across quartiles. Although the cutoffs increase linearly in score, the unequal width of quartiles, particularly the broader range in Q1 (26–43) and Q4 (53–70), may obscure a true dose-response pattern. This inconsistency could also reflect heterogeneity in food sources contributing to uPDI or may represent a chance finding.

Several biological pathways may explain the observed inverse association between hPDI and liver fat content. Diets characterized by higher intakes of fruits, vegetables, whole grains, legumes, and nuts provide abundant dietary fiber^36^, polyphenols^37^, and unsaturated fatty acids, all of which have been inversely associated with liver fat accumulation^38^ by enhancing insulin sensitivity, attenuating hepatic lipogenesis, and exerting anti-inflammatory and antioxidant effects.^39–41^ Notably, in our food group analysis, higher intake of nuts was significantly associated with lower odds of MASLD and reduced liver fat content, consistent with prior literature linking nut consumption to lower prevalence of NAFLD in U.S. adults.^42^ These constituents may also influence the gut-liver axis by promoting a favorable gut microbiota composition, thereby reducing endotoxemia and hepatic inflammation associated with higher hepatic adiposity.^43–45^ Moreover, healthful plant-based dietary patterns are typically low in refined carbohydrates, added sugars, and red or processed meats, components associated with increased hepatic fat accumulation through mechanisms including oxidative stress, mitochondrial dysfunction, and systemic inflammation^46,47^. Collectively, these nutritional attributes may contribute to the reduced liver fat content observed among individuals with greater adherence to healthful plant-based diets.

A notable strength of our study is the inclusion of a racially and ethnically heterogenous population, which enabled a more representative assessment of the associations between dietary patterns and MASLD, while also allowing for the characterization of potential differences in susceptibility across groups. We identified a statistically significant interaction between hPDI and ethnicity for liver fat content, indicating that the relationship between healthful plant-based dietary patterns and liver outcomes may vary across ethnic groups. Stratified analyses revealed the strongest inverse associations among Latino and White participants, with a suggestive but borderline-significant association in Japanese Americans. While the hPDI quartile cutoffs for Japanese and White participants were similar in score range, Japanese Americans had fewer individuals per quartile and slightly less variability in scores, particularly at the upper end of the distribution. This reduced dispersion may have limited power to detect significant associations despite a comparable effect size to that observed in Whites. These findings align with prior research from the APS and MEC cohorts, which have demonstrated that susceptibility to NAFLD and hepatic fat accumulation differs by ethnicity, even after accounting for total adiposity.^35,48^

Other strengths of this study include the use of MRI—a gold-standard noninvasive imaging technique— to quantify liver fat, as well as the application of validated plant-based diet indices. Additionally, comprehensive adjustment for demographic, lifestyle, and clinical variables enhances the robustness of the observed associations.

This study has also several limitations. First, its cross-sectional design limits causal inference. Second, dietary intake was self-reported and, thus, subject to measurement error and, possibly, recall bias although participants were in good general health. Lastly, sample sizes in some racial/ethnic subgroups were relatively small, which may have led to limited power for stratified analyses.

## Conclusion

In summary, adherence to a healthful plant-based dietary pattern was associated with significantly lower liver fat content and reduced odds of MASLD. These findings highlight the importance of diet quality in mitigating liver fat accumulation and suggest that healthful plant-based diets may serve as an effective strategy for MASLD prevention across diverse populations. Our results carry important clinical implications: they reinforce the need for dietary guidance that goes beyond “plant-based” labels to emphasize nutrient density and food quality, and they point to the potential benefit of culturally tailored nutrition interventions to optimize prevention strategies. Finally, these findings support broader efforts to promote dietary pattern-based approaches to liver disease prevention, especially in light of few approved pharmacologic therapies. Future longitudinal studies are warranted to establish causality and evaluate the effectiveness of tailored dietary interventions.

## Supplementary Material

This is a list of supplementary files associated with this preprint. Click to download.

- SupplementaryFigureS1.png
- SupplementaryTableS1.docx
- SupplementaryTableS2.docx
- SupplementaryTableS3.docx
- SupplementaryTableS4.docx

## Figures and Tables

**Table 1:** Participant characteristics according to extreme quartiles of the plant-based diet indices, the Multiethnic Cohort Adiposity Phenotype Study (n = 1,598).^1^

	Overall	PDI		hPDI		uPDI	
	(n = 1598)	Q1 (n = 477)	Q4 (n = 343)	Q1 (n = 538)	Q4 (n = 280)	Q1 (n = 387)	Q4 (n = 421)
Age, y, (mean ± SD)	69.2 ± 2.7	68.9 ± 2.7	69.55 ± 2.6	68.9 ± 2.8	69.3 ± 2.7	69.2 ± 2.7	69.1 ± 2.8
Female, n (%)	825 (52%)	254 (53%)	177 (53%)	274 (51%)	145 (52%)	196 (51%)	221 (52%)
Ethnicity, n (%)								
African American	274 (17%)	67 (14%)	68 (20%)	118 (22%)	42 (15%)	45 (12%)	90 (21%)
Native Hawaiian	255 (16%)	106 (22%)	37 (11%)	108 (20%)	36 (13%)	64 (17%)	63 (15%)
Japanese American	402 (25%)	116 (24%)	83 (24%)	114 (21%)	82 (29%)	106 (27%)	91 (22%)
Latino	345 (22%)	77 (16%)	86 (25%)	101 (19%)	53 (19%)	69 (18%)	114 (27%)
White	322 (20%)	111 (23%)	69 (20%)	97 (18%)	67 (24%)	103 (27%)	63 (15%)
Smoking status, n (%)								
Never smoker	1,002 (63%)	295 (62%)	230 (67%)	327 (61%)	172 (61%)	241 (62%)	278 (66%)
Former smoker	596 (37%)	182 (38%)	113 (33%)	211 (39%)	108 (39%)	146 (38%)	143 (34%)
BMI, kg/m^2^	27.9 ± 4.8	28.9 ± 5.0	26.8 ± 4.7	29.0 ± 4.9	25.9 ± 4.4	27.3 ± 4.5	28.4 ± 4.8
Physical activity, METs/d	1.5 ± 1.4	1.3 ± 1.2	1.8 ± 1.7	1.35 ± 1.2	1.75 ± 1.6	1.7 ± 1.45	1.4 ± 1.4
Alcohol intake, g/d	4.0 ± 6.5	4.3 ± 6.9	3.4 ± 5.7	3.7 ± 6.2	4.4 ± 6.8	4.3 ± 6.5	3.3 ± 6.0

1 Values are presented as mean ± standard deviation unless noted for number (percentage). Participants were grouped into quartiles (Q1–Q4) based on their score, with Q1 representing the lowest adherence and Q4 the highest.

BMI = body mass index; MET = metabolic equivalents. Percentages may not total 100% due to rounding.

**Table 2:** Association between plant-based diet indices and liver fat content.

Index	N (%)	Adjusted Mean (% volume)	95% CI	p-value
PDI				
Q1 (< 44)	477 (30)	6.23	(5.80, 6.69)	
Q2 (44–48)	476 (30)	6.01	(5.59, 6.45)	0.20
Q3 (48–52)	302 (19)	5.87	(5.46, 6.31)	0.09
Q4 (≥ 64)	343 (21)	5.77	(5.34, 6.22)	0.06
hPDI				
Q1 (< 45)	538 (34)	6.52	6.06, 7.01	
Q2 (44–48)	415 (26)	6.19	5.76, 6.64	0.17
Q3 (48–52)	365 (23)	5.96	5.55, 6.40	**0.02**
Q4 (≥ 70)	280 (17)	5.39	5.01, 5.80	**0.001**
uPDI				
Q1 (< 45)	387 (24)	5.67	5.28, 6.09	
Q2 (44–48)	441 (28)	6.23	5.81, 6.69	**0.02**
Q3 (48–52)	349 (22)	5.75	5.35, 6.18	0.74
Q4 (≥ 70)	421 (26)	6.42	5.96, 6.91	**0.002**

Adjusted mean liver fat values and 95% confidence intervals were estimated from multivariable linear regression models. Models were adjusted for age, sex, BMI, physical activity, alcohol intake, smoking status, race/ethnicity, and included a sex × BMI interaction term. Quartile 1 (Q1) served as the reference group. Statistically significant results (*p* < 0.05) are shown in bold. CI = confidence interval for the mean; PDI = overall plant-based diet index; hPDI = healthful plant-based diet index; uPDI = unhealthful plant-based diet index.

**Table 3:** Association between plant-based diet indices and MASLD, in odds ratios (ORs) and 95% confidence intervals (CIs).

Index	OR (95% CI)	p-value	p-trend
PDI			
Q1 (< 45)	1.00		
Q2 (44–48)	0.86 (0.62, 1.18)	0.35	
Q3 (48–52)	0.83 (0.60, 1.15)	0.25	
Q4 (≥ 64)	0.09 (0.64, 1.26)	0.53	0.56
hPDI			
Q1 (< 45)	1.00		
Q2 (44–48)	0.81 (0.58, 1.12)	0.19	
Q3 (48–52)	0.75 (0.54, 1.04)	0.09	
Q4 (≥ 70)	0.58 (0.41, 0.81)	**0.002**	**0.002**
uPDI			
Q1 (< 45)	1.00		
Q2 (44–48)	1.23 (0.88, 1.70)	0.22	
Q3 (48–52)	0.97 (0.70, 1.36)	0.88	
Q4 (≥ 70)	1.38 (0.99, 1.92)	0.06	0.13

Models adjusted for age, sex, race/ethnicity, BMI, physical activity. Statistically significant results (*p* < 0.05) are in bold. CI = Confidence Interval; hPDI = healthful plant-based diet index; uPDI = unhealthful plant-based diet index; PDI = overall plant-based diet index.

## Abbreviations

MASLD: Metabolic Dysfunction-Associated Steatotic Liver Disease
NAFLD: Non-Alcoholic Fatty Liver Disease
APS: Adiposity Phenotype Study
MEC: Multiethnic Cohort Study
QFFQ: Quantitative Food Frequency Questionnaire
PDI: Overall Plant-Based Diet Index
hPDI: Healthful Plant-Based Diet Index
uPDI: Unhealthful Plant-Based Diet Index
NASH: Non-Alcoholic Steatohepatitis
FLI: Fatty Liver Index
HCC: Hepatocellular Carcinoma
MPED: MyPyramid Equivalents Database
MRI: Magnetic Resonance Imaging
DXA: Dual-Energy X-ray Absorptiometry
BMI: Body Mass Index
MET: Metabolic Equivalent
OR: Odds Ratio
